# The mTOR/ULK1 signaling pathway mediates the autophagy-promoting and osteogenic effects of dicalcium silicate nanoparticles

**DOI:** 10.1186/s12951-020-00663-w

**Published:** 2020-08-31

**Authors:** Wang Ruolan, Chen Liangjiao, Shao Longquan

**Affiliations:** 1 Nanfang Hospital, Southern Medical University, Guangzhou, 510515 China; 2 Guangdong Provincial Key Laboratory of Construction and Detection in Tissue Engineering, Guangzhou, 510515 China; 3 Key Laboratory of Oral Medicine, Guangzhou Institute of Oral Disease, Stomatology Hospital of Guangzhou Medical University, Guangzhou, 510140 China

**Keywords:** Dicalcium silicate, Osteogenesis, Autophagy, MTOR/ULK1

## Abstract

A novel bioactive inorganic material containing silicon, calcium and oxygen, calcium silicate (Ca_2_SiO_4_, C_2_S) with a CaO-SiO_2_ ingredient, has been identified as a potential candidate for artificial bone. Autophagy has an essential function in adult tissue homoeostasis and tumorigenesis. However, little is known about whether silicate nanoparticles (C_2_S NPs) promote osteoblastic differentiation by inducing autophagy. Here we investigated the effects of C_2_S NPs on bone marrow mesenchymal stem cell differentiation (BMSCs) in osteoblasts. Furthermore, we identified the osteogenic gene and protein expression in BMSCs treated with C_2_S NPs. We found that autophagy is important for the ability of C_2_S NPs to induce osteoblastic differentiation of BMSCs. Our results showed that treatment with C_2_S NPs upregulated the expression of BMP2, UNX2, and OSX in BMSCs, and significantly promoted the expression of LC3 and Beclin, while P62 (an autophagy substrate) was downregulated. C_2_S NP treatment could also enhance Alizarin red S dye (ARS), although alkaline phosphatase (ALP) activity was not significantly changed. However, all these effects could be partially reversed by 3-MA. We then detected potential signaling pathways involved in this biological effect and found that C_2_S NPs could activate autophagy by suppressing mTOR and facilitating ULK1 expression. Autophagy further activated β-catenin expression and promoted osteogenic differentiation. In conclusion, C_2_S NPs promote bone formation and osteogenic differentiation in BMSCs by activating autophagy. They achieve this effect by activating mTOR/ULK1, inducing autophagy, and subsequently triggering the WNT/β-catenin pathway to boost the differentiation and biomineralization of osteoblasts.

## Introduction

Injuries, trauma, tumors, and other bone diseases are the major causes of bone defects and thus pose immense challenges for orthopedic and reconstructive surgeons. In addition, with the increase in the aging population, age-related bone loss is an urgent problem to be solved. The development of bone substitute materials provides good perspective towards bone defect management [1]. In recent years, it has been found that silicon-containing bioactive materials stimulate cell proliferation, activate the expression of osteogenic genes, and demonstrate a favorable capacity for osteogenesis both in vitro and in vivo [2]. As an important ingredient of Portland cement, dicalcium silicate (Ca_2_SiO_4_, C_2_S) is also an essential element in the calcium-silicate system, and it has good prospects for bone defect regeneration. In general, it is acknowledged that a precondition for the binding of both materials and living bone tissue is the establishment of a bone-like hydroxyapatite layer, which is an indication of biological activity [3]. Additionally, with favorable reproducibility, biocompatibility, leakproofness, and antimicrobial potential, dicalcium silicate has been shown to trigger the secretion of cytokines by osteoblasts, as well as to possess the ability to promote hard tissue formation [4–6]. Prior research has demonstrated the promising biological activity, biocompatibility, and mechanical performance of γ-Ca_2_SiO_4_ ceramics and their ability to support mesenchymal stem cell adhesion and spreading. Another study has demonstrated that C_2_S-released silicon ions are essential in bone repair [7–9]; thus, C_2_S might be a promising bone implant material.

Although the rise of various new bone replacement materials has provided new hope for bone defect repair, the mechanism(s) underlying this process are poorly understood. In the context of normal development, autophagy, as a key intracellular degradation and recycling process, has an essential role in the maintenance of homeostasis and remodeling of cells. Autophagy allows cells to deliver cytoplasmic components to the lysosome and then degrade and recycle unwanted or abnormal organelles and impaired macromolecules [10, 11]. More recently, autophagy has been shown to contribute significantly to the preservation of the differentiation capacity of stem cells, and it can also maintain bone homeostasis and protect BMSCs from oxidative stress [12–14]. Autophagy can also positively mediate osteogenesis of stem cells and osteoblasts. Research has shown that in undifferentiated cells, autophagosomes do not fuse with lysosomes, while in differentiating mesenchymal stem cells (MSCs), autophagy is activated during early differentiation [14]. While autophagy participates in osteoclast mineralization and bone homeostasis, there are reports that autophagic vacuoles serve as carriers for releasing the mineralized substrates [15]. Inhibition of autophagy could result in a reduction of bone quality in mice, which suggests that autophagy may be a vital contributor to the preservation of bone mass and osteoclast mineralization in vitro as well as in vivo [10, 16]. Some recent research has demonstrated that particular silica nanoparticles exhibit intrinsic probiotic activity against osteogenic and osteoclastic cells [15, 17]. Thus, in our study we speculated that autophagy is triggered by the C_2_S NPs in BMSCs, and C_2_S NPs then promote BMSC osteogenesis through the activation of autophagy.

Prior work has supplied evidence supporting the contribution of AMPK activation to the regulation of the differentiation and mineralization of osteoblasts in a variety of manners, such as mTOR and ULK1. As a critical mechanism responsible for cartilage generation and osteogenesis, more recent research has largely concentrated on the mammalian rapamycin target protein (mTOR) signaling pathway [18, 19]. Studies have also shown that AMPK may induce autophagy by directly activating ULK1 or inhibiting mTOR [15]. Activation of the AMPK/ULK1-dependent autophagy can be correlated positively with osteogenic differentiation of MSCs [20, 21], and ULK1 overexpression or rapamycin can activate autophagy and then notably elevate the osteoblast differentiation activity and restore the bone volume [22].

In the present research, we explored the impact of C_2_S nanoparticles on osteoblastogenesis, and the autophagy function of BMSCs treated with C_2_S NPs and the effect on differentiation. We further explored whether activation of ULK1 downstream of the mTOR pathway could enhance osteoblast differentiation and mineralization by inducing autophagy.

## Materials and methods

### Preparation and characterization of C_2_S NPs

#### Preparation of C_2_S NPs

C_2_S particles of 99% purity were synthesized by the sol–gel process in the laboratory of Shanghai Institute of Ceramics, Chinese Academy of Sciences.

#### Characterization of C_2_S NPs

Using a scanning electron microscope (SEM; Nova Nano SEM 430, FEI, Finland) combined with energy dispersive X-ray spectroscopy (DX-4 system, EDAX, USA), the particle morphology was identified. An investigation of the crystal structure was performed with X-ray diffraction (XRD; Geigerflex, Rigaku Corporation, Japan), and the diffraction pattern was determined over a range of 2θ from 10°–80° with monochromatic Cu Kα radiation. Apart from the application of tetrasodium phosphate and a surfactant, with vigorous stirring (pumping at 80 rpm and 100 rpm cycles), an ultrasonic bath was utilized to disperse the particles. The particle size distributions that were measurable in the 64 channels ranged from around 0.1 to 150 μm; the ultimate size distributions were identified using Fraunhofer calculations. An atomic force microscope (AFM) (Agilent Technologies, Inc. USA) was applied to inspect the microstructure. Furthermore, a Fourier transform infrared spectrometry (FTIR) approach (Thermo Fisher Scientific Inc. USA) was conducted to analyze the chemical composition.

### Cell culture

Cyagen Bioscience provided bone marrow mesenchymal stem cells (BMSCs), which were cultured in Minimum Essential Medium Eagle – α modification (α-MEM, Gibco, USA) supplemented with 10% FBS and streptomycin (Gibco; Thermo Fisher Scientific, USA) and 100 μg/mL penicillin, in a 5% CO_2_ atmosphere at 37 °C. Incubation of the first 7 passages of BMSCs was utilized for the following experiments, wherein all the media were replaced at 2-day intervals.

In the original α-MEM, the cell viability, proliferation, and cytoskeleton assembly were assayed. The cellular osteogenic differentiation mediated by osteogenic medium containing 10% FBS, 50 mg/ml ascorbic acid, 10 nM dexamethasone, and 10 mM b-glycerophosphate, was measured by alkaline phosphatase (ALP) activity, collagen secretion, and extracellular matrix (ECM) mineralization.

### ICP-MS elemental analysis

To assess the durability of C_2_S NPs in growth medium, dissolution studies of C_2_S were performed in complete medium. C_2_S NPs were incubated in medium for 24 h. After incubation, a 0.22-μm filtration membrane was utilized to filter the suspension, and the filtered medium was collected. Inductively coupled plasma-mass spectrometry (ICP-MS; Thermo Fisher Scientific, USA) was used to determine the calcium, silicon, and phosphorus concentrations.

### Cell proliferation assay

#### Cell counting kit-8 (cck-8) assay

In 96-well plates, BMSCs were incubated with C_2_S NPs of 0, 10, 50 and 100 μg/mL at a thickness of 6 × 10^3^/cm^2^ for 6, 12, and 24 h, at 3 days and 7 days. The cellular activity was determined using the cell counting kit-8 (CCK-8). After 100 µL of complete medium with 10 µL of cell counting kit-8 solution (Dojindo Molecular Technologies, Japan) were pumped into each well, the cells were cultured at 37 °C for 1 h [23]. To obtain optical density (OD) values, the absorbance peak at 450 nm was identified with a microplate meter (Molecular Device, USA).

#### Lactate dehydrogenase (LDH) assay

The effect of C_2_S NPs on cell membrane was assessed by studying the leakage of LDH from cells. In brief, a total of 6 × 10^3^ BMSCs were seeded in 96-well plates and subsequently treated with C_2_S NP samples at concentrations of 0, 10, 50, and 100 μg/mL over 6, 12, and 24 h, at 3 days and 7 days. Medium collected from each well was centrifuged, and a microplate reader (Molecular Device, USA) was employed to measure the absorption at 490 nm.

### Flow cytometry assay

#### Cell cycle analysis

A Cell Cycle and Apoptosis Analysis Kit (Beyotime, China) was used to investigate the cell cycle. Following treatment with various concentrations of C_2_S NPs for 24 h, BMSCs were subsequently collected and rinsed three times with PBS. After fixing the BMSCs in cold (4 °C) 75% ethanol overnight, they were rinsed three times with PBS and stained with sodium propyl iodide (PI) in 37 °C RNase for 30 min. Flow cytometry (Millipore, Billerica, MA) was employed to assess all the samples.

#### Cell apoptosis analysis

Annexin V-fluorescein isothiocyanate (FITC) and PI were utilized to identify apoptosis. The Annexin V-FITC Apoptosis Detection Kit (Beyotime, China) was also utilized to determine the percentages of apoptotic and necrotic cells. In short, following treatment with C_2_S NP sequence dilutions (0, 10, 50, and 100 μg/mL), BMSCs were subsequently acquired at 24 h and 7 days and then rinsed with PBS. Based on the recommendations of the manufacturer, samples were dyed with Annexin V-FITC and PI before assessment by flow cytometry (BD FACSAria III, USA).

### Confocal microscopy

#### Cell morphology

On glass coverslips, BMSCs were sown at a thickness of 2 × 10^5^/cm^2^ in 6-well plates and incubated with C_2_S NPs at 0, 10, 50 and 100 μg/mL for 24 h. Following PBS washes, fixation with 4% cis-paraformaldehyde, dialysis with 0.5% Triton X-100, and blockade with 1% BSA, the samples were subsequently dyed with DAPI and rhodamine phosphatidylcholine, and observed by confocal microscopy (Leica SP8, Germany).

#### Immunofluorescence assay

BMSCs treated with various concentrations of C_2_S NPs for 3, 6, and 12 h were coated overnight with 4% paraformaldehyde at 4 °C, permeabilized with 0.01% Triton X-100 for 20 min at room temperature, and blocked with 5% BSA for 30 min at 37 °C. After incubation with specific primary antibody LC3 (1 µg/ml Abcam, UK) overnight at 4 °C and treatment with fluorescent secondary antibodies (1:50; Cell Signaling Technology (USA)), all the specimens were rinsed and stored at room temperature for 1 h. The nucleus was counterstained with DAPI (Biofroxx, Germany) for 5 min at room temperature and then washed three times with PBS. The dyed cells were examined under a confocal microscope (Leica SP8, Germany). ImageJ software (NIH) was utilized to analyze cell images, where the width, perimeter, length, and area of the cells were determined, and the roundness (4π*Area/[perimeter]2) and aspect ratio (length/width) was calculated. Each sample was randomly selected under 3 fields of view analyzing 3 samples per group.

### Transmission electron microscopy (TEM)

TEM was utilized to examine cell adhesion and uptake. Using a concentration of 0 and 50 µg/mL C_2_S NPs for 6 h, the BMSCs were inoculated in 10 cm^2^ cell culture dishes for 6 h, 1 day, 3 days, and 7 days. After immobilizing the cultured cells in 0.1 M malic acid buffer with 2.5% glutaraldehyde, the cells were fixed with 1% osmium tetroxide. Following ethanol dehydration, infiltration of the cells, and final embedding in Epon resin, ultrathin sections were counterstained with lead citrate and 4% urea acetate and observed under a high-resolution HT7700 transmission electron microscope (Hitachi Scientific Instruments, Japan).

### Alkaline phosphatase activity

At a density of 1 × 10^4^/cm^2^, the cells were seeded in 48-well plates and treated with a range of C_2_S NPs at 0, 10, 50 and 100 μg/mL for 3, 7, and 14 days. Osteogenesis regulated by autophagy was observed, which was induced by C_2_S NPs. BMSCs were treated with 0 μg/mL C_2_S NPs, 5 mM 3-methyladenine (3-MA), 10 nM rapamycin, 50 μg/mL C_2_S NPs, 50 μg/mL C_2_S NPs +3-MA, and 50 μg/mL C_2_S NPs + rapamycin for 7and 14 days. Staining and colorimetric methods were used to determine the ALP activity of BMSCs. With regard to ALP staining, samples were rinsed three times with PBS and fixed with 4% cis-butenal for 12 min, followed by staining with the BCIP/NBT alkaline phosphatase staining kit (Beyotime, China). Subsequently, a stereomicroscope (Leica EZ4HD, Germany) was used to photograph the samples following a PBS rinse.

### Mineralization

Within the standard culture media and at a density of 1  ×  10^4^ cells/cm^2^, the samples were seeded in 48-well plates and incubated and treated with 0, 10, 50 and 100 μg/mL C_2_S NPs for 7 and 14 days. We also detected osteogenesis regulated by autophagy induced by C_2_S NPs. BMSCs were treated with 0 μg/mL C_2_S NPs, 5 mM 3-MA, 10 nM rapamycin, 50 μg/mL C_2_S NPs, 50 μg/mL C_2_S NPs +3-MA, and 50 μg/mL C_2_S NPs + rapamycin for 7, 14, and 21 days. By salicylin red staining, extracellular mineralization was determined. After rinsing three times with PBS, the cells were immobilized with 4% cis-butenal for 15 min and then incubated with 0.1% salicylin red S (pH 4.1 Solarbio, China) at room temperature for 3 min. Thereafter, the specimens were washed with ddH_2_O and were photographed under a stereomicroscope (Leica EZ4HD, Germany).

### Quantitative real-time PCR assay

Gene expression was detected by qPCR. BMSCs (3  ×  10^5^ cells) were cultured with 0, 10, 50 and 100 μg/mL C_2_S NPs in 6-well plates for 3, 7, and 14 days. Following isolation of total RNA with TRIzol and quantification with a Nano-Drop 2000 system (Thermo Fisher Scientific, USA), cDNA was reverse transcribed from each sample in accordance with the specifications of the Prime Script RT kit (Takara, Japan). The SYBR green inLightCycler480 Sequence Detector System (Takara, Japan, Roche, Switzerland) was utilized to perform the real-time PCR. The relative mRNA expression was calculated based on the quantification of ΔΔCt [24]. Genes associated with osteogenesis, which include type I collagen (COLI), runt-associated transcription factor 2 (RUNX2), osterix (OSX), and bone morphogenetic protein 2 (BMP2) were identified on days 3, 7 and 14 Normalization to the gene expression of GAPDH was performed. Primer BLAST. Primer BLAST from PubMed online was used to design the primers, which were then synthesized by Sangon Biotech (China). See Table 1 for the primers used for qPCR.

**Table 1:** Primers used for real-time PCR

Mtot	Primer
COL1	5′-CCAGCGAAGAACTCATACAG-3′
	5′-GAGCGAAGGGTCAGTCAG-3′
OSX	5′-GAGCAAAGTCAGATGGGTAAG-3′
	5′-CACCAGGTCCAGGCAACA-3′
BMP2	5′-GAATGACTGGATCGTGGCACCTC-3′
	5′-GGCATGGTTAGTGGAGTTCAGGTG-3′
RUNX2	5′-CCCAGCCACCTTTACCTACA-3′
	5-TATGGAGTGCTGCTGGTCTG-3′
GAPDH	5′-TGTCGTGGAGTCTACTGGTG-3′
	5′-GCATTGCTGACAATCTTGAG-3′

### Western blot analysis

#### Osteogenic protein analysis

In 6-well plates, BMSCs were sown at a density of 3 × 10^5^ cells and treated with C_2_S NPs at 0, 10, 50 and 100 μg/mL for 3, 7, 14, and 21 days. In addition, we detected osteogenesis regulated by autophagy induced by C_2_S NPs. BMSCs were treated with 0 μg/mL C_2_S NPs, 5 mM 3-MA, 10 nM rapamycin, 50 μg/mL C_2_S NPs, 50 μg/mL C_2_S NPs +3-MA, and 50 μg/mL C_2_S NPs + rapamycin for 7 and 14  days. After scratching the adherent cells from the culture dish and rinsing three times with ice-cold PBS, RIPA lysis buffer (Haimen Beotem, China) was used to extract the total protein from the cell pellet. After incubating for 30 min at 4 °C, the specimens were subsequently centrifuged for 20 min at 14,000*g*. The supernatant was transferred, mixed, and boiled in SDS sample buffer (6.8, 2% SDS, 10% glycerol, and 62.5 mM Tris–HCl pH). The BCA kit (Beyotime, China) was applied to determine the protein concentration. After loading onto 10% SDS-PAGE gels with equal amounts of total protein, and transferring to PVDF membranes (General Electric, USA), the samples were intubated for 1 h at room temperature with TBS-T (25 mM Tris, pH 7.4, 150 mM NaCl, and 0.1% Tween-20) supplemented with 5% skimmed dry milk, followed by incubation with primary antibody at 4 °C overnight. Upon 3 washes with TBS-T, goat anti-rabbit IgG HRP-linked antibodies (Beyotime, China) were incubated at an ambient temperature for 1 h. The plaques were identified with an ECL detection kit (General Electric, USA) in accordance with the manufacturer’s protocol. Incubation of protein samples at 3, 7, and 14 days was detected with the following antibodies: anti-GAPDH (1:2000), anti-BMP2 (1:1000; Ab Cam, USA); anti-OPN (1:2000), and anti-RUNX2 (1:2000; Ab Cam, USA). To analyze the results, the relative ratio was determined using ImageJ software.

#### Autophagy protein analysis

Western blotting was conducted to determine the expression of molecules associated with the autophagic pathway, as described earlier. BMSCs were sown in 6-well plates at a density of 3  ×  10^5^ cells and then treated with 0 μg/mL C_2_S NPs, 5 mM 3-MA, 10 nM rapamycin, 50 μg/mL C_2_S NPs, 50 μg/mL C_2_S NPs +3-MA, and 50 μg/mL C_2_S NPs + rapamycin for 7 and 14 days. Protein extraction and SDS polyacrylamide electrophoresis were carried on as previously mentioned. Specimen protein was tested with the following antibodies: anti-GAPDH (1:2000), anti-Beclin (1:2000 Ab Cam, USA), anti-P62 (1:2000; Ab Cam, USA), and anti-LC3 (1:1000; Ab Cam USA). To analyze the results, the relative ratio was determined using ImageJ software.

### In vivo analysis

Southern Medical University Animal Center (Guangzhou, China) provided the SD rats (n = 6, weight = 250 g). All experiments associated with animals were approved by and conducted in accordance with the regulations of the China National Ethics Committee on Animal Welfare, which issued the approval number L2018033. Experimental rats were kept in a ventilated animal chamber at a temperature of 23 ± 1 °C and 55–65% relative humidity, under a 12-h light/dark cycle. According to the method described previously, a mouse model of renal tubular deficiency was utilized to assess the osteogenic activity of C_2_S NPs [25]. Briefly, surgery was performed under general anesthesia. A 5-mm-diameter calvarial defect was generated in each SD rat. The defects received no graft in the control group and were treated with C_2_S NPs in the C_2_S group.

To measure the parameters of skeleton formation, intraperitoneal injections of tetracycline (5 mg/kg; Sigma-Aldrich) and calcein (25 mg/kg; Sigma-Aldrich) were dosed at 2 weeks and 1 week before sacrifice to mark newly formed bone surfaces. At week 4, 8, and 12 following implantation, the rats were sacrificed, and buffered glutaraldehyde (4%)-glutaraldehyde (0.25%) fixative (pH 7.4) was utilized to fix the calvariae at 4 °C for 3 days, which was applied to assess bone regeneration. The samples were further analyzed using micro-CT and histological techniques.

#### Observation of new bone formation

Calvarial bone was collected, dehydrated (from 75% to 100%) with increasing ethanol concentrations, and embedded in polymethyl methacrylate (PMMA). The central region of the sagittal defect was sliced to an ultimate thickness of approximately 40 μm. Excitation/emission wavelengths of 488/500–550 nm (green carbenicillin, green) and 543/580–670 nm (tetracycline, red) were labeled using a fluorescence microscope (Olympus, Japan). The marking distance between carbenicillin green and tetracycline was divided by the time interval of the marking period. Then rate of mineral adhesion was obtained. Van Gieson’s picrofuchsin and Stevenel’s blue dye were adopted to qualitatively observe the formation of new bone.

#### Micro-CT evaluation of bone formation

Micro-computed tomography (micro-CT) was utilized to investigate new bone formation at 4, 8, and 12 weeks with parameters of 8.85 μm/pixel of resolution, accompanied by 50 kV of voltage and 500 μA current. The commercial software MIMICS (NRecon, USA; CTvox, USA) was used to assess a fixed global threshold of 20%. The new bone volume was identified as the total volume of the 5-mm-diameter bone disc centered on the defect.

#### Histological analysis

After 72 h in 4% cis-butylene solution, all specimens were fixed and decalcified in 10% ethylenediaminetetraacetic acid (EDTA) and stored for 25 days at 4 °C. After the calcium was eliminated, the skull was dehydrated, cleaned, prepared in paraffin blocks in accordance with standard histological procedures, and sliced at 5 μm. Dyeing of histological Sects. (8 μm) for the target of interest was performed. Hematoxylin and eosin (H&E) staining were performed thereafter. For BMP2, LC3, p-ULK1, and ULK1 immunochemical staining, the sections were stained with the following primary antibodies: anti-LC3 (1:500; Ab Cam USA), anti-BMP2 (1:500; Ab Cam USA), anti-ULK1 (1:500, Ab Cam USA), and antip-ULK1 (1:500; CST USA). Peroxidase assays were performed using the Vectastain ABC kit with DAB substrate kit. ImageJ was used to quantify the area of newly formed bone tissue.

### Statistical analyses

Experimental results are displayed as the mean ± SEM in vitro and mean ± SD in vivo of a minimum of three independent trials. Inter-group difference statistical significance was assessed with the GraphPad Prism (GraphPad, CA, USA) Student’s t test and one-way ANOVA (Tukey’s multiple comparison test). *p < 0.05 was considered statistically significant.

## Results

### Characterization of C_2_S NPs

**Fig. 1:**
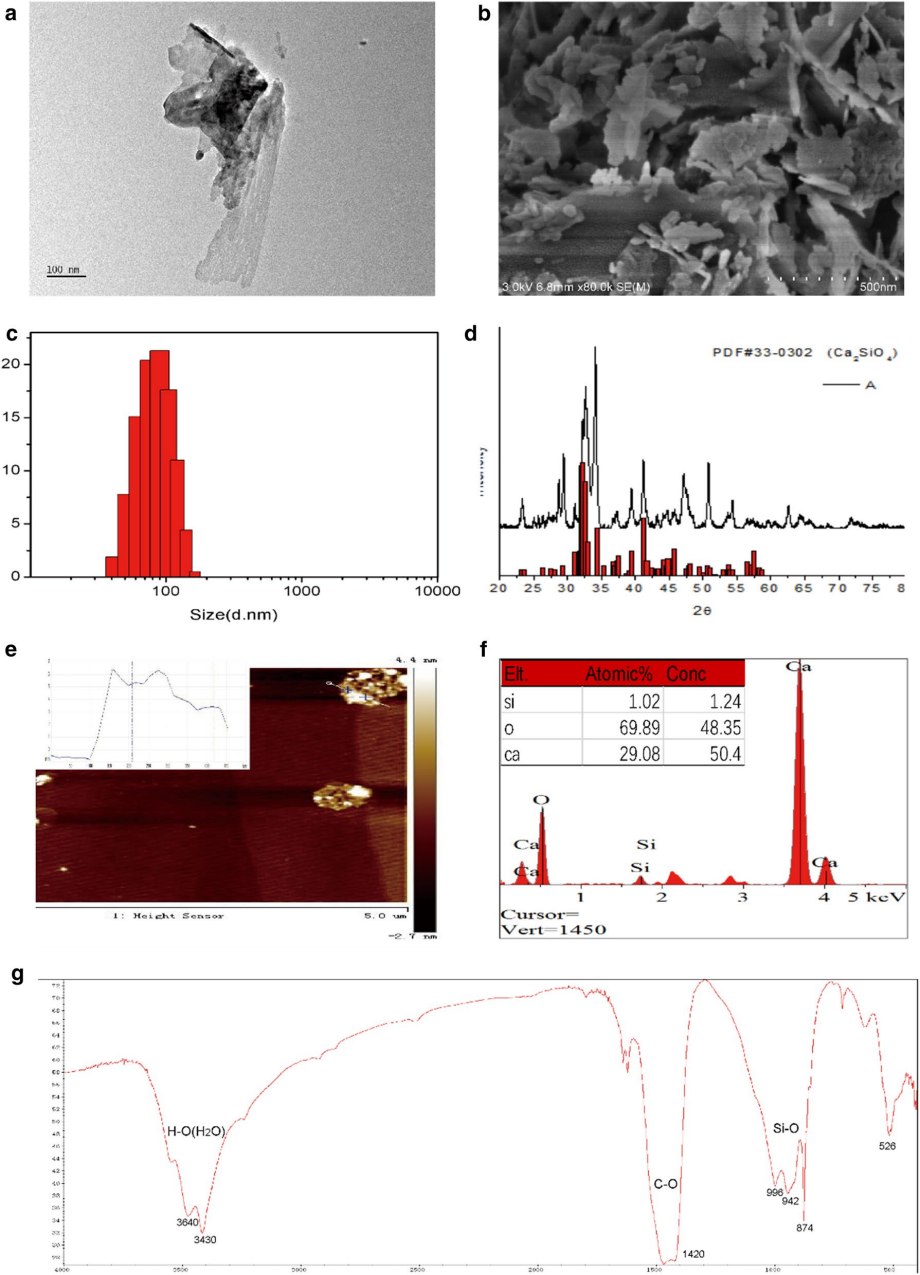
Physicochemical characterization of C_2_S NPs.** a** Representative TEM image of the C_2_S NP morphology and size.** b** Representative SEM image of C_2_S NP morphology.** c** Particle size of C_2_S powder ranged from 40-150 nm in diameter by laser diffraction.** d** XRD analyses indicated that the spectrum of sample corresponded to Ca_2_SiO_4_.** e** Representative AFM image and thickness analysis of C_2_S NPs.** f** EDS analyses detected Ca, Si, and O corresponding to Ca_2_SiO_4_.** g** Functional groups of C_2_S NPs identified by the FTIR spectrum showed the presence of C-O, Si–O and unbound water

The TEM and SEM images of the C_2_S nanoparticle samples revealed that the particles of C_2_S powder had a size under 100 nm (Fig. 1a) and irregular shapes and surfaces (Fig. 1b). The particle size of the C_2_S powder was visualized between 40 and 150 nm using laser diffraction, and 50% of the particles in the sample were less than 100 nm (Fig. 1c). The spectra of the samples were shown to be consistent with those of Ca_2_SiO_4_ by XRD analysis. The corresponding peak and the standard peak were matched (Fig. 1d). The mean thickness was approximately 6.0 nm via AFM (Fig. 1e). EDS analyses identified Ca, Si, and O, and the atomic ratio of Si, O, and Ca was 1.02, 60.89, and 29.08, corresponding to Ca_2_SiO_4_. The sample contained C_2_S NPs (Fig. 1f). The FTIR spectra (Fig. 1g) showed absorption peaks at 615, 800–1000, 1420, and 3400–3700, confirming the presence of C-O, Si–O and unbound water, which are characteristic vibrations of C_2_S NPs.

### Biocompatibility evaluation of C_2_S NPs

#### Dissolution of C_2_S NPs in complete cell culture medium

**Fig. 2:**
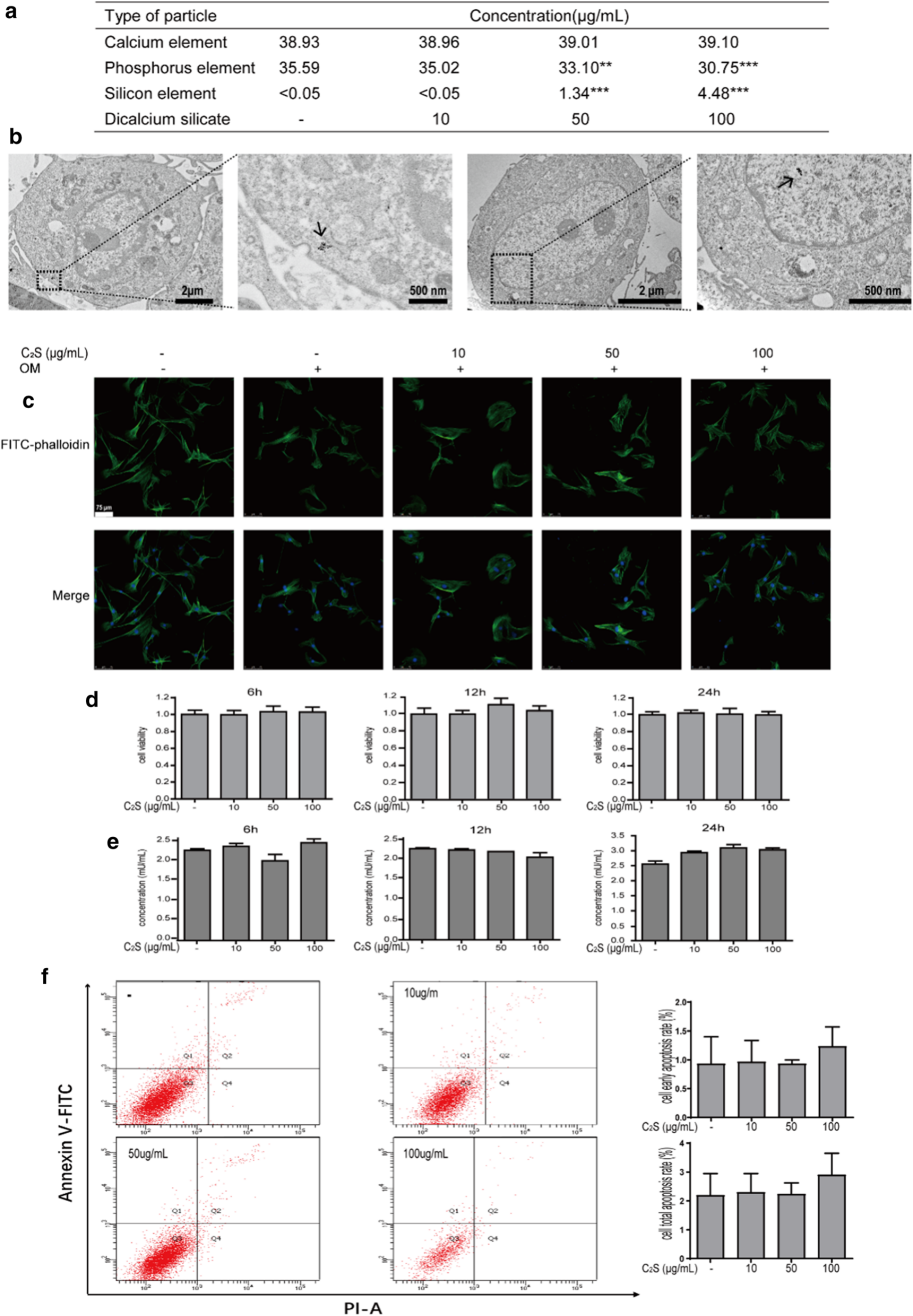
Biocompatibility evaluation of BMSCs exposed to C_2_S NPs (0, 10, 50, 100 μg/mL).** a** ICP-MS exploration of ion levels released from C_2_S NPs in extracellular fluid for 24 h.** b** Uptake of C_2_S NPs by BMSCs was observed via TEM at 6 h.** c** Cellular morphology and structures of cell organelles were observed by rhodamine-phalloidin (Green) and DAPI (Blue) staining of BMSCs at different times points (6, 12, and 24 h), scale bar 20 µm.** d** Cell viability was evaluated using CCK-8 assays at different times points (6, 12, and 24 h).** e** The levels of LDH release were detected in BMSCs post-treatment with C_2_S NPs for 6, 12, and 24 h;** f** Cells were stained with Annexin V-FITC and PI and analyzed by flow cytometry. Quantification of the cell early and total apoptosis ratio is shown below on the right. Values are expressed as the mean ± SEM n = 3. * p < 0.05, ** p < 0.01, *** p < 0.001 compared with the control group

For the purpose of exploring the ion levels released by C_2_S NPs in the extracellular fluid. the concentrations of silicon and calcium in various concentrations of complete media were assessed (Fig. 2a). The different concentrations of C_2_S NPs put up dissolution after incubation for 24 h. Along with the increased concentration of C_2_S NPs, the concentration of silicon ions was found to be dramatically elevated (*p *< 0.05). However, the percentage of composition of calcium ions was not meaningfully altered with the increase in C_2_S NPs. In addition, the concentration of phosphorus was significantly decreased. The maximum concentration of silica was approximately 4.48 μg/mL at a saturation of 100 μg/mL C_2_S NPs, and the lowest silica saturation was under 0.05 μg/mL at a saturation of 10 μg/mL C_2_S NPs; at a C_2_S NP concentration of 50 μg/mL, the silica concentration was approximately 1.34 μg/mL. At 10 μg/mL C_2_S NPs, the saturation of calcium was nearly 38.96 μg/mL. The calcium ion concentration was 39.01 μg/mL when the C_2_S NP concentration was 50 μg/mL, and at 100 μg/mL C_2_S NPs, the concentration of calcium ions was 39.10 μg/mL. The concentration of phosphorus was significantly decreased with increasing concentrations of C_2_S NPs. The highest concentration of phosphorus was 35.02 μg/mL at a concentration of 10 μg/mL C_2_S NPs, while the lowest saturation at 100 μg/mL C_2_S NPs was 30.75 μg/mL; at a concentration of 50 μg/mL, the phosphorus ion concentration was 33.10 μg/mL.

#### Uptake of C_2_S NPs by BMSCs

One important issue suggesting a possible biological response from NPs is cellular uptake. With exposure to C_2_S NPs for 6 h, 24 h, 3 days, and 7 days, the uptake of C_2_S NPs by BMSCs was observed via TEM. As shown in Fig. 2b, Additional file 1: S1a, C_2_S NPs were observed both in cytoplasm and nuclei nanoparticles. Moreover, there were no significant effects of exposure to C_2_S NPs on cell morphology and organelle structure in BMSCs (Fig. 2c).

#### Cell viability and apoptosis profiles of C_2_S NPs

All experiments had cell survival rates greater than 95% (both pre and post powder exposure). The CCK-8 assay was adopted to assess cell survival in all experiments. Cell viability. Following exposure of BMSCs to C_2_S NPs for 6, 12, 24 h and 7 days, no significant cytotoxicity was detected. Cells were viable at over 99% in all trial groups (Fig. 2d). With increasing concentrations, there was a slight increase in the cell viability of BMSCs, but there was no significant difference. A lactate dehydrogenase (LDH) leakage assay was performed to validate the results of the CCK-8 viability assessment (Fig. 2e, Additional file 1: S1b). The results showed that C_2_S NPS caused no significant changes in cell membrane permeability. Apoptosis was detected by flow cytometry utilizing the FITC Annexin V Apoptosis Detection Kit. The results showed no obvious apoptosis in BMSCs treated with C_2_S NPS. Approximately 95% of BMSCs in the C_2_S NPs group did not undergo apoptosis (Fig. 2f, Additional file2: S2c). In the C_2_S NPs group, the apoptosis rates slightly increased, but the difference between the C_2_S NPS-treated group and the control group was not significant. These results indicated that BMSCs treated with different concentrations of C_2_S NPs did not cause obvious cytotoxicity.

### Osteogenic effect of C_2_S NPs

#### C_2_S NPs promote cell proliferation of BMSCs

**Fig. 3:**
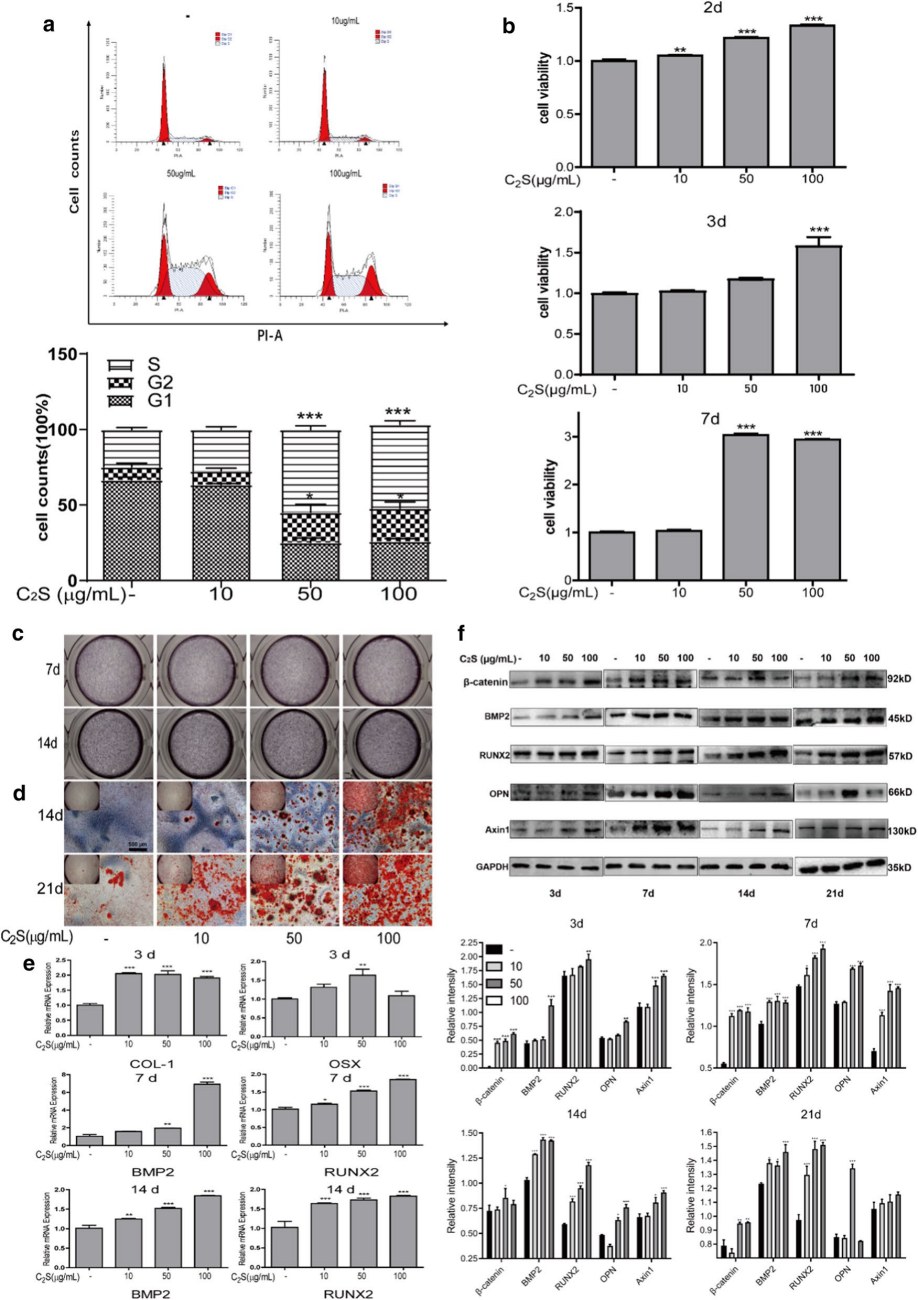
Osteogenic effect of C_2_S NP (0, 10, 50, 100 μg/mL) nanoparticles in vitro.** a** The cell cycle was detected by flow cytometry. BMSCs were incubated with 50 and 100 μg/mL C_2_S NPs for 24 h.** b** Cell proliferation was evaluated using CCK-8 assays at different times points (2, 3, and 7 days). ALP activity (**c**) and the formation of mineralized nodules (**d**) were detected by BCIP/NBT and Alizarin red S staining accompanied by increasing concentrations of C_2_S NPs at different time points (7, 14, 21 days).** e** The total mRNA levels of COLI, OSX (Osterix), RUNX2, and BMP2 in BMSCs treated with C_2_S NPs (0, 10, 50, 100 μg/mL) at different time points (3, 7, 14, 21 days) was detected by RT-PCR.** f** The total protein levels of β-catenin, RUNX2, BMP2, OPN and AXIN1 were detected by western blotting, and the cells were treated as in (**e**). Densitometry data were normalized to GAPDH. The relative optical density was analyzed using ImageJ software (below). Values are expressed as the mean ± SEM. n = 3. *p < 0.05, **p < 0.01, ***p < 0.001. Compared with the control group

Cell cycle profiling (Fig. 3a) illustrated that the proportion of cells in G1 phase dropped from 66.58% to 48.48% and 46% in cells treated with 50 μg/mL and 100 μg/mL C_2_S NPs, whereas the proportion of cells in S phase increased significantly from 24.74% to 48.09% and 45.53% (*p* < 0.05).The cell cycle results showed that BMSCs incubated with C_2_S NPs promoted cell cycle changes from G1 phase to S phase, which indicated an increase in cell division. The CCK-8 assay was utilized to determine cell proliferation, which confirmed the increasing proliferation of BMSCs treated with C_2_S NPs. As shown in Fig. 3b, BMSCs were exposed to C_2_S NPs for 2, 3, and 7 days, and the proliferating cells in each group continued to increase with increasing concentrations of C_2_S NPs. At each time point, we found significantly increased cell proliferation when BMSCs were treated with 50 μg/mL and 100 μg/mL C_2_S NPs (*p* < 0.05). However, BMSCs treated with 10 μg/mL C_2_S NPs showed no significant increase in cell proliferation (*p* > 0.05).

#### Mineralization of BMSCs treated with C_2_S NPs

For the exploration of BMSCs early and late osteogenic cell differentiation, ALP activity was investigated. ALP is an important early-stage osteoblastic marker, and its activity was evaluated following the incubation of cells with osteobd)last-inducing conditional media and different concentrations of C_2_S NPs for 7, 14, and 21 days. As shown in Fig. 3c, there was no significant difference in ALP activity between the C_2_S NP treatment groups and the control group after 7 and 14 days (*p* > 0.05). ALP activity was increased slightly with increasing C_2_S NP concentrations after culture for 4 days (*p* > 0.05). These results indicated that C_2_S NPs did not promote BMSC ALP expression. It was also an important indicator of late differentiation of osteoblasts, demonstrating that osteoblasts had developed into organic matter for bone formation by entering the mineralization phase. The formation of mineralized nodules (Fig. 3d) was utilized to assess the effects on the late osteogenesis of BMSCs of C_2_S NPs. Mineralized nodules were observed by ARS staining at 14 and 21 days after osteogenic differentiation. The accumulation of mineral matrix deposition increased as the color of the stain darkened and thickened with increasing concentration of C_2_S NPs. These results indicated that C_2_S NPs could facilitate mineralization.

#### The effect of C_2_S NPs on the expression of osteogenic genes and proteins

mRNA expression of the osteogenesis-regulated genes COLI, OSX (Osterix), RUNX2, and BMP2 was identified by RT-PCR. On the third day, COLI and OSX were significantly increased after treatment with 10, 50, and 100 μg/mL C_2_S NPs. BMP2 and RUNX2 mRNA expression significantly increased at 7 and 14 days (Fig. 3e). We identified the protein expression related to osteoblast differentiation, and the results showed that C_2_S NP stimulation accelerated osteoblast differentiation of BMSCs. Increased protein expression of β-catenin, Runx2, and BMP2 was detectable at days 3, 7, 14, and 21, Axin1 expression was increased at days 3, 7, and 14, and OPN expression was increased at days 7 and 14; At day 21, the differences in protein expression began to decline (Fig. 3f).

### C_2_S NPs stimulated autophagy in BMSCs

**Fig. 4:**
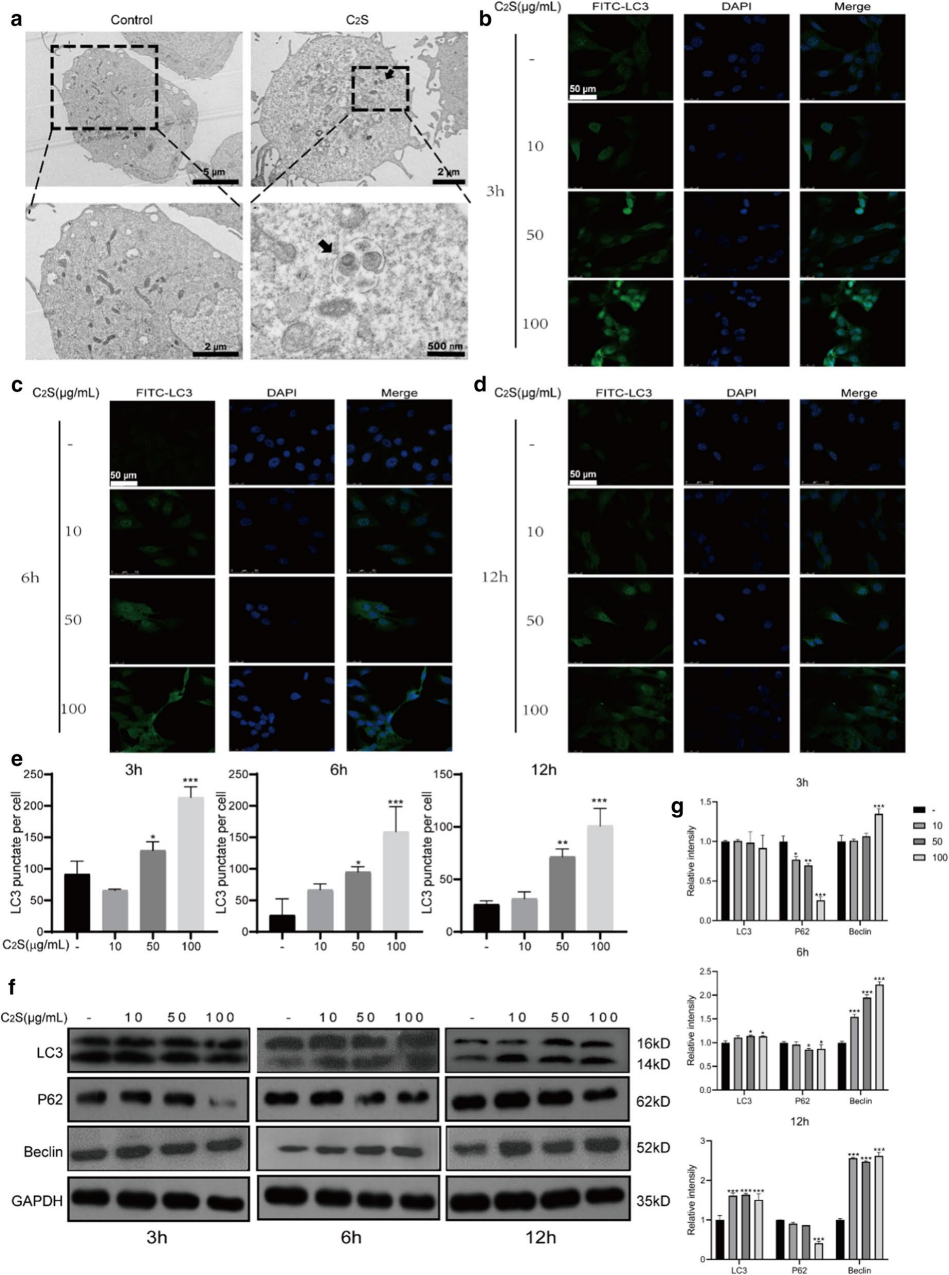
C_2_S NPs stimulate autophagy in BMSCs (**a**) TEM observations of BMSCs following treatment with 50 μg/mL C_2_S NPs for 24 h. Distinct autophagic vacuoles (black arrows) were denoted in the enlarged images (lower) from the dash line squares.** b–d** BMSCs were treated with C_2_S NPs (0, 10, 50, 100 µg/mL) for 3, 6, and 12 h. FITC-labeled LC3 was observed under a fluorescence microscope.** e** The fluorescence intensity ratio was analyzed using ImageJ software (below).** f** total protein levels of Beclin, P62, and LC3(LC3II/LC3I) detected by western blot analysis, treating the cells as in (d). g Densitometry data were normalized to GAPDH. The relative optical density was analyzed using ImageJ software. Values are expressed as the mean ± SEM, n = 3. *p < 0.05, **p < 0.01, ***p < 0.001 compared with the control group

TEM was utilized to study the subcellular effect of C_2_S in BMSCs. Our TEM images revealed considerable autolysosomes and autophagosomes in BMSCs treated with C_2_S NPs compared with the control BMSCs (Fig. 4a, Additional file 1: S1A). Immunofluorescence results also confirmed the occurrence of autophagy in BMSCs. LC3 accumulation was detected with a confocal microscope. Treatment of C_2_S NPs resulted in a marked increase in FITC-labeled LC3B formation. We observed that LC3B formation increased under C_2_S NPs treatment at 3, 6, and 12 h (Fig. 4b–e). To explore whether autophagy could be induced by C_2_S NPs in BMSCs, we next detected changes in several autophagy-related proteins. A known biomarker of autophagosome formation is the transformation of the mature form of LC3β-I into the LC3β-II form associated with cleaved/lapidated autophagosome, while P62 is a biomarker of autolysis degradation. As presented in Fig. 4f, g, osteoblast induction dramatically increased the conversion of LC3β-II from LC3β-I according to Western blotting, and the autophagy cargo P62 was decreased upon autophagy activation in C_2_S NP-treated cells, indicating increased autophagic activity. Treatment of BMSCs with C_2_S NPs for 3, 6, and 12 h resulted in a strong increase in LC3β-II. Likewise, we detected an increase in beclin1, which also had a critical function in the autophagy configuration.

### C_2_S NPs promote osteogenesis through the autophagy effect

**Fig. 5:**
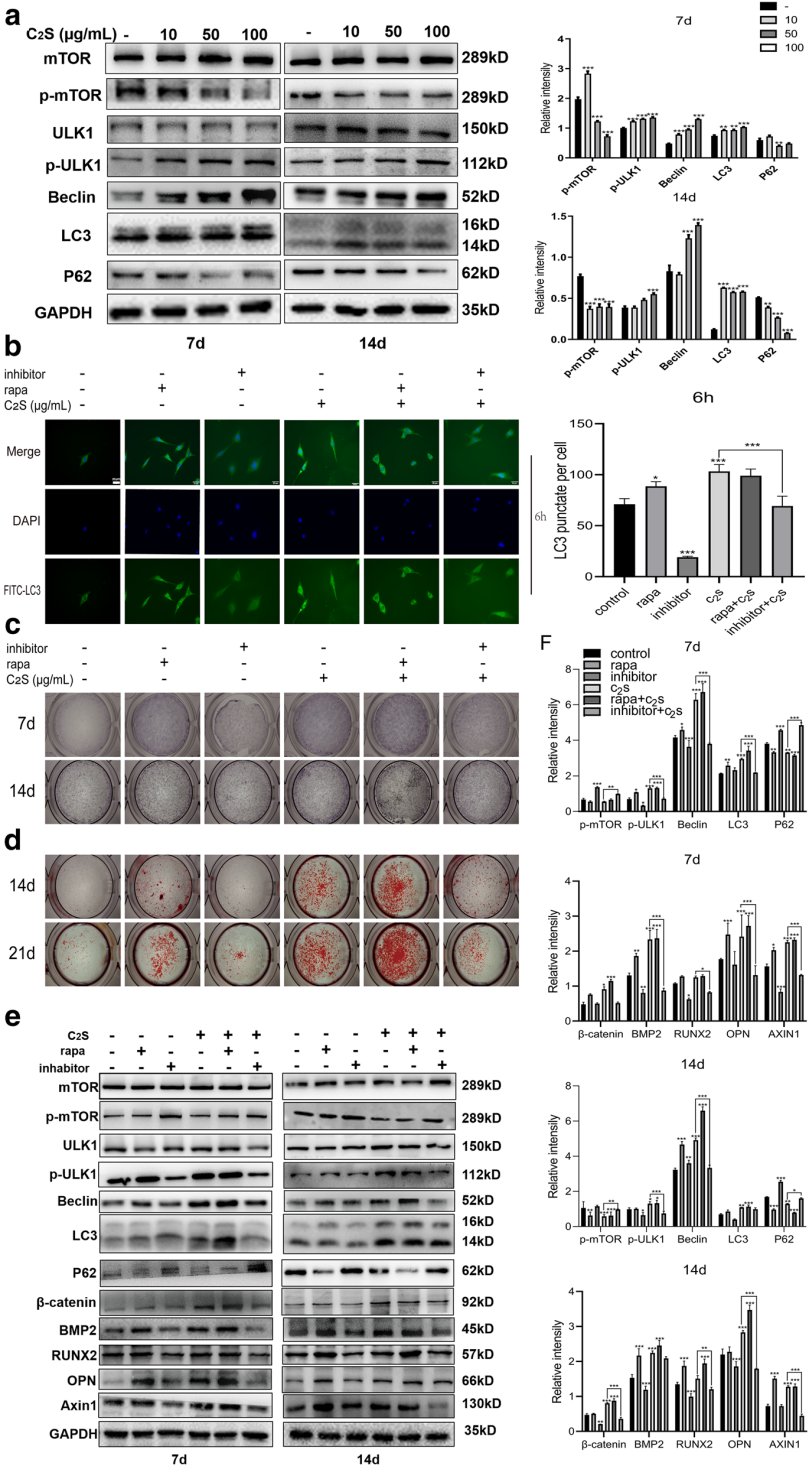
C_2_S NPs promote osteogenesis through an autophagy effect in vitro.** a** The total protein levels of mTOR, p-mTOR, ULK1, p-ULK1, Beclin, P62, and LC3 in BMSCs treated with C_2_S NPs (0, 10, 50, 100 μg/mL) at different time points (7 and 14 days) were detected by western blot analysis, and densitometry data were normalized to GAPDH. The relative optical density was analyzed using ImageJ software (below).** b** BMSCs were treated with 3-MA, rapamycin, C_2_S NPs (50 μg/mL), C_2_S NPs + 3-MA, and C_2_S NPs + rapamycin for 6 h. FITC-labeled LC3 was observed under a fluorescence microscope. The fluorescence intensity ratio was analyzed using ImageJ software (below). ALP activity (**c**) and the formation of mineralized nodules (**d**) were detected by BCIP/NBT and Alizarin red S staining, accompanied by 3-MA, rapamycin, C_2_S NPs (50 μg/mL), C_2_S NPs + 3-MA, and C_2_S NPs + rapamycin at different time points (7, 14 days). ** f** Total protein levels of mTOR, p-mTOR, ULK1, p-ULK1, Beclin, P62, LC3 β-catenin, RUNX2, BMP2, OPN, and Axin1 in BMSCs were detected by western blot analysis. Inhabitor + C_2_S group compared with C_2_S group.** f** Densitometry data were normalized to GAPDH. The relative optical density was analyzed using ImageJ software. Values are expressed as the mean ± SEM. n = 3. *p < 0.05, **p < 0.01, ***p < 0.001. compared with the control group

**Fig. 6:**
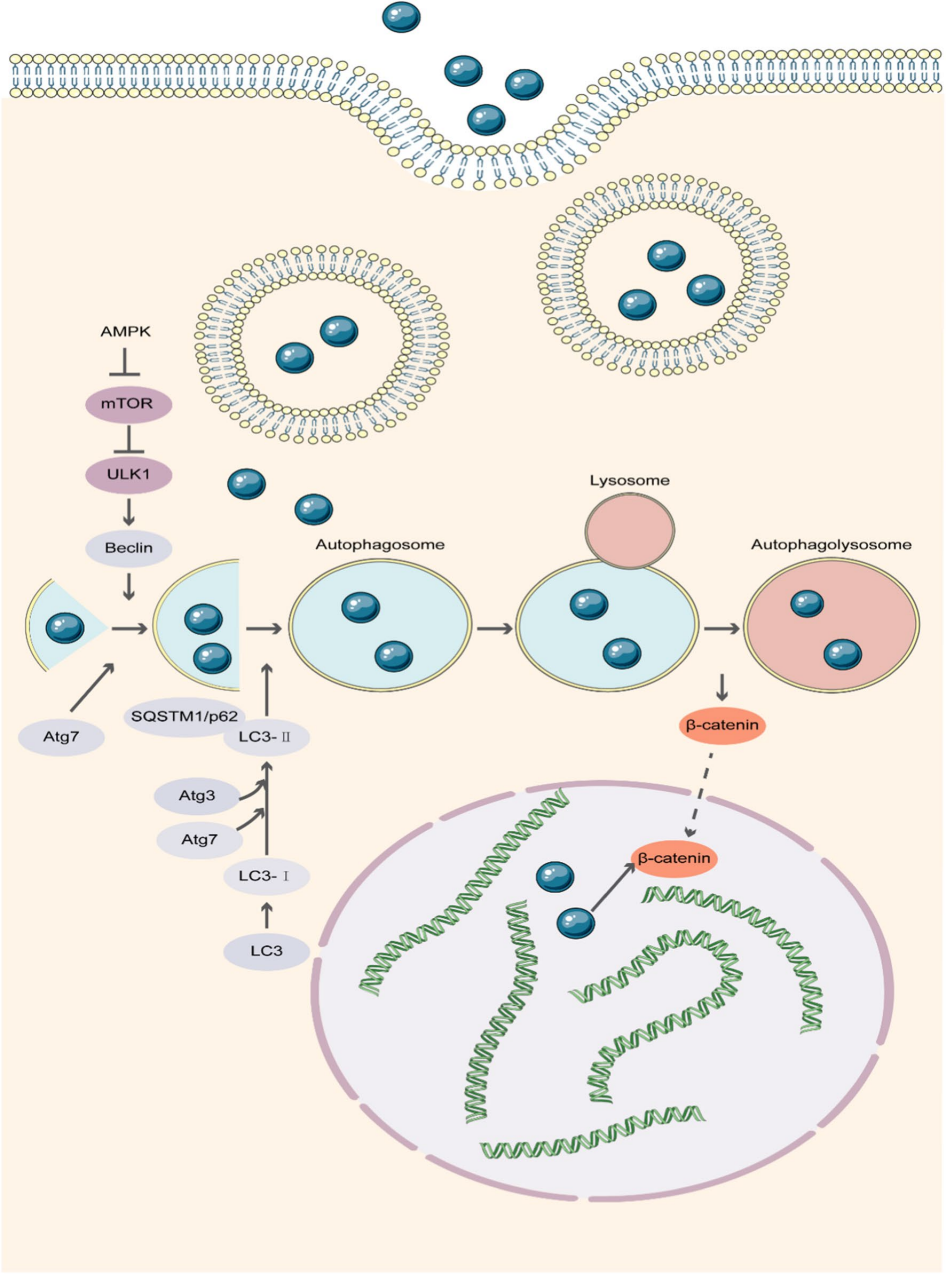
Signaling pathway by which C_2_S NPs promote osteogenesis through an autophagy effect. C_2_S NPs can enter the cell and be trafficked to autophagosomes to activate autophagy and further promote the osteogenic differentiation of MSCs in vitro and in vivo. C_2_S NPs can activate the mTOR/ULK1 signaling pathway to induce autophagy and activate the Wnt/β-catenin pathway to promote osteogenesis. C_2_S NPs can promote osteogenesis by activating autophagocytosis via the mTOR/ULK1 pathway

We then examined whether C_2_S NPs could continuously induce autophagy. The Western blot results revealed increased protein expression associated with autophagy. C_2_S NPs promoted activation of autophagy, which was manifested by elevated rates of conversion from LC3-I to LC3-II, enhanced generation of serine/threonine protein kinase ULK1 and beclin, and reduced expression of P62 at days 7 and 14 compared with the control group (Fig. 5a). To explore whether autophagy participates in regulating osteogenic differentiation induced by C_2_S NPs, the autophagy inhibitor 3-MA and the activator rapamycin were applied. The 3-MA significantly inhibited C_2_S NP-induced autophagy activation, while rapamycin enhanced autophagy (Fig. 5b). We first evaluated the acceleration of osteoblast differentiation in response to 3-MA and rapamycin treatment. Compared with the control treatment, 3-MA reduced while rapamycin accelerated osteoblast differentiation of BMSCs. Following culture with 3-MA and rapamycin treatment, ALP and alizarin red staining were applied to determine osteoblast differentiation. As illustrated in Fig. 5c, NBT-formazan stained with BCIP/NBT working fluid was markedly elevated in the rapamycin group, whereas the enhancement of osteogenic differentiation induced by C_2_S NP incorporation was reduced in the 3-MA group at 7, 14, and 21 days after 3-MA pretreatment. Correspondingly, while autophagy occurred, the alizarin red staining results indicated the formation of more mineralized nodules with autophagy, and 3-MA inhibited the formation of mineralized nodules (Fig. 5d). The levels of Runx2 and BMP2, which are markers of osteogenesis, could be significantly reduced by pretreatment with 3-MA prior to osteogenic induction according to the Western blot results. In addition, the activation of autophagy triggered by C_2_S NPs was markedly inhibited by 3-MA (Fig. 5e, f). Conversely, pretreatment with the autophagy activator rapamycin elevated the levels of Runx2 and BMP2 (Fig. 5e, f). These findings suggested that osteogenic differentiation could be enhanced by autophagy. C_2_S NP administration regulated autophagic activation. The role of autophagy in these procedures is being investigated further in our current research. As revealed in Fig. 5e, f, C_2_S NP treatment significantly decreased the p-mTOR/mTOR ratio and increased the p-ULK1/ULK1 ratio, which is a typical autophagy activity mediator. Nevertheless, C_2_S NPs also enhanced β-catenin, together with P62 degradation. These results provided evidence that autophagy could be mediated by C_2_S NPs through suppression of mTOR and promotion of ULK1 activation. In summary, we concluded that BMSC differentiation could be mediated by C_2_S NPs administration through activation of mTOR/ULK1-induced autophagy (Fig. 6).

### C_2_S NPs promote osteogenesis through the autophagy effect in vivo

**Fig. 7:**
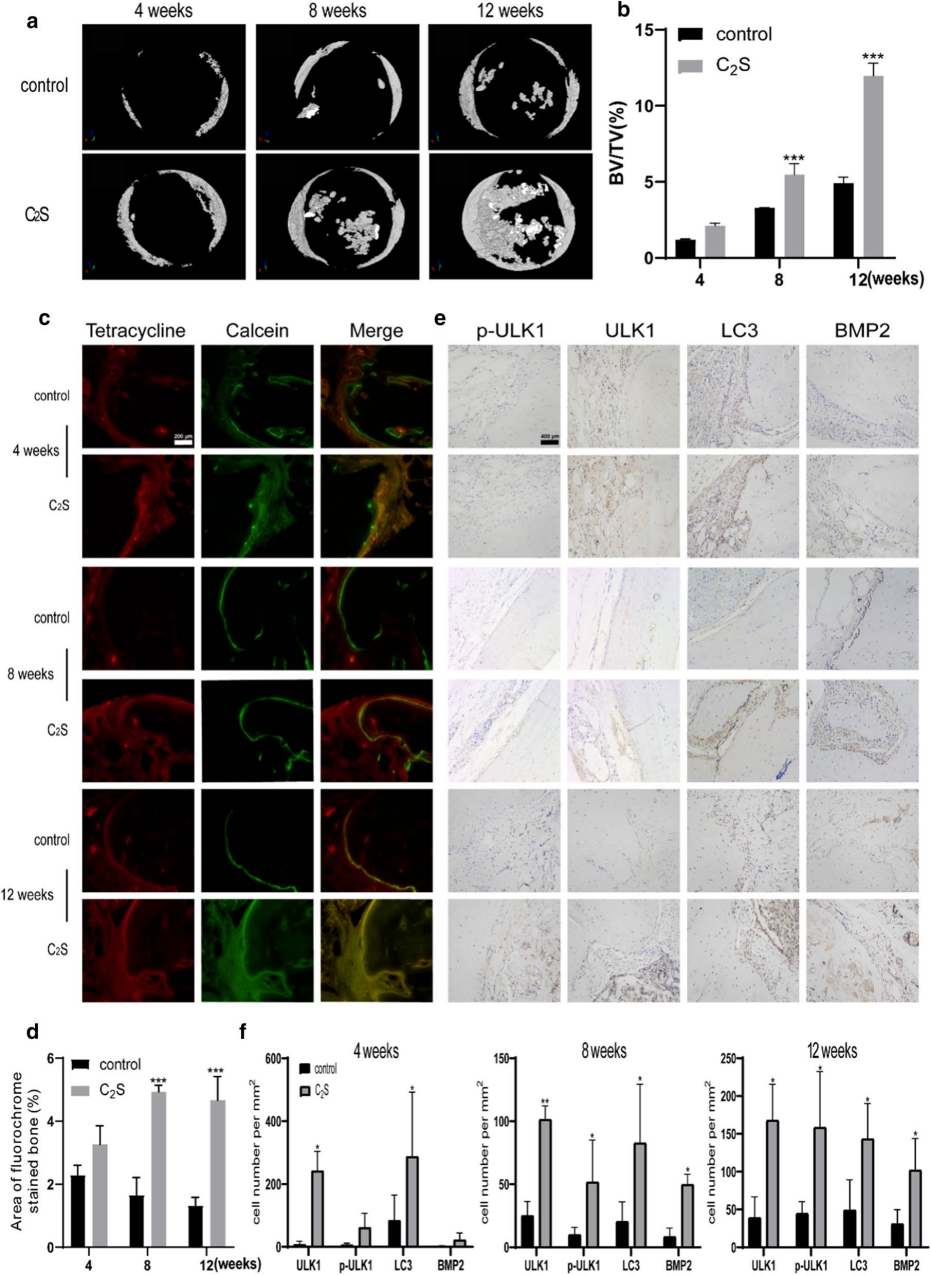
C_2_S NPs promote osteogenesis through an autophagy effect in vivo.** a** Micro-computed tomography (micro CT) reconstruction images revealed new bone formation in the defects implanted with C_2_S NPs at 4, 8, and 12 weeks.** b** The relative optical density of new bone formation in the defects was analyzed using ImageJ software.** c** Histological images of newly formed bone with C_2_S NPs at 4, 8, and 12 weeks after surgery.** d** The area between the centers of the red band (tetracycline) and the green band (calcein) in the defects was analyzed using ImageJ software.** e** Immunohistochemical analysis of the protein expression of BMP2, LC3, ULK1, and p-ULK1in rat calvarial defects after implantation of C_2_S NPs at 4, 8, and 12 weeks.** f** The relative optical area of new bone formation in the defects was analyzed using ImageJ software. Values are expressed as the mean ± S.D. n = 3. * p < 0.05, **p < 0.01, ***p < 0.001 compared with the control group

We employed an osteoclast-deficient model to investigate the role of autophagy in osteoclast differentiation. No animal deaths or significant body weight changes were caused by C_2_S NPs, and HE staining revealed no significant inflammatory cell infiltration in the defect area (Additional file 2: Fig. S2b, c), indicating no obvious toxicity. Compared with the control group, C_2_S NPs led to abundant bone formation, as evidenced by the elevated ratio of bone volume to tissue volume (BV/TV). It can be concluded that much more new bone formed in (Fig. 6) defects implanted with C_2_S NPs compared with the control subjects through Micro-CT reconstruction (Fig. 7a). Quantitative analysis showed that the relative new bone mass was considerably greater in the C_2_S NPs group than the control group (Fig. 7b). Van Gieson’s picrofuchsin and Stevenel’s blue staining were performed to evaluate the bone formation (Additional file 2: Fig. S2d), and double-labeling immunofluorescence staining (Fig. 7c, d) was performed. The MAR was calculated as the area between the centers of the red band (tetracycline) and the green band (calcein). As shown in Fig. 7f, markedly higher MAR was observed at 4, 8, and 12 weeks in the C_2_S NP group compared with the control group (n = 6, *p* < 0.05). Furthermore, BMP2 immunohistochemical staining of histological sections revealed elevated bone matrix deposition and increased expression of osteogenic markers in the C_2_S NPs group, suggesting a significantly enhanced activating effect of C_2_S NPs on osteogenic cell differentiation (Fig. 7e, f). C_2_S NPs facilitated the osteogenic process of adherent cells in vivo, which was consistent with in vitro experimental results. With the aim of validating whether the boosted activation of autophagy was attributed to bone formation, we assayed autophagy using immunohistochemical methods. The C_2_S NPs had a significant promoting effect on the activation of autophagy base don ULK, LC3, and p-ULK1 staining. The results showed that the number of cells positively stained for LC3, ULK1, and p-ULK1 was greater in the C_2_S NPs treatment group than the control group (Fig. 7e, f). This result is consistent with the activation of autophagy by C_2_S NPs, which activated the expression of ULK1 in the calvaria defect area. These observations indicated that C_2_S NPs could promote osteogenesis and mineralization by activating autophagy in the bone defect area.

## Discussion

Dicalcium silicate is an amorphous silicate-based material that is a component of bioactive glasses, has good biocompatibility, and can form a chemical bond with bone tissue; therefore, it is often used in bone substitute materials, artificial bone dust, and potential realistic materials for dental implants and orthopedic treatments [4, 26–28]. Because it is composed of natural minerals, it has a wide range of sources and is easy to obtain, so it is a very economical material. Due to its good biological activity, dicalcium silicate is considered a bioactive material with good application prospects.

Nanomaterials such as silica nanoparticles are often used in biomaterials due to their sizable specific surface area, biocompatible capacity, stability, and outstanding optical and electrical characteristics[29, 30]. Our study showed that dicalcium silicate nanoparticles (C_2_S NPs) are irregularly shaped nanoparticles with a hydrated particle size of approximately 100 nm; therefore, they have a large specific surface area. C_2_S NPs can release a small number of silicon ions in complete culture medium.

In addition to these physicochemical properties, C_2_S NPs have good cytocompatibility. Previous studies have shown that most nanoparticles, such as silica-based nanoparticles, enter cells by endocytosis [16, 31]. Our TEM results also revealed that C_2_S NPs could be ingested by BMSCs and enter the cytoplasm and nucleus, where they further produce a series of biological effects. However, we did not observe an obvious vesicle structure around the nanoparticles Thus, we speculate that C_2_S NPs first adhered to the cell membrane, and then the C_2_S NPs nanoparticles were internalized and encircled by regions of the plasma membrane, growing within the cell and forming vesicles consisting of absorbed C_2_S nanoparticles. The EDS results showed that the C_2_S NPs contained calcium, silicon, and other biologically active groups that could promote proliferation and BMSC osteogenic differentiation in vitro and promote the formation of new bone in the bone defect area in vivo. The results of a previous study are consistent with our hypothesis, which found that the silicon component could be released from bioactive glass implants, mediating the generation of a hydroxyapatite layer containing a calcium-deficient surface that further interacts with collagen fibrils of damaged bone and subsequently allows the bioactive glass to bond to the surrounding tissue [32]. In our research, BMSCs treated with C_2_S NPs could promote mineralization and osteogenic differentiation and upregulate the osteogenic genes OSX, COL1, RUNX2, BMP2, and OPN, as well the osteogenic proteins Runx2, BMP2, OPN. Interestingly, however, C_2_S NPs did not promote the expression of ALP in BMSCs compared with the control group. This result is different from a previous study that found that treatment of BMSCs with silica-based nanomaterials could promote the expression of ALP [32, 33]. The difference may be due the use in our control group of osteoblast-inducing conditional medium, as well as dicalcium silicate nanoparticles instead of dicalcium silicate extract. ALP expression is promoted by osteoblast-inducing conditional medium, and C_2_S NPs do not further promote ALP expression under these conditions. In addition, bone formation may be facilitated by the characteristics of the particles themselves rather than by ions in the solution. C_2_S NPs may provide an ingredient for osteogenic mineralization, directly promoting the mineralization of BMSCs, rather than promoting the deposition of collagen by activating the expression of ALP and then promoting the mineralization of BMSCs [34].

In vivo, we found that mice treated with C_2_S NPs showed more new bone generation in the bone defect area than the control group. The potential mechanism by which C_2_S NPs promote osteogenic differentiation and mineralization in vitro and in vivo was examined, We found that C_2_S NPs could activate autophagy in BMSCs. Previous studies have found that autophagy plays an important role in osteogenic differentiation. The balance of autophagy appears to be vital for efficient MSC differentiation and function. Therefore, we investigated whether C_2_S NPs could promote osteogenic mineralization by activating autophagy [14]. Our TEM results revealed that C_2_S NPs could be taken up by cells. Previous studies have shown that nanomaterials can be taken up by cells and then combine with some proteins to form a structure called a protein crown. Protein crowns may be considered a misfolded protein. They are then further encapsulated and degraded by autophagosome encapsulation [35]. Next, the autophagosome binds to the lysosome to form an acidic environment, and the acidic environment of the autolysosome can promote C_2_S NP release of calcium and silicon to promote biomineralization [36]. Our study found that C_2_S NPs could activate the expression of LC3, as well as beclin, Atg3, and Atg7, while degrading P62. In vivo experiments also confirmed the occurrence of autophagy, and the results of immunohistochemistry showed that the expression of LC-3 was upregulated in the defect area of the C_2_S NPs group compared with the control group. Thus, the C_2_S NPs could promote autophagy without hindering the progression of autophagic flow and be degraded by autophagy. Autophagy can degrade damaged organelles and foreign bodies to form phosphate, which is secreted into the cytoplasm to provide an ingredient for and promote biomineralization [37].

To verify whether C_2_S NPs could promote osteogenesis and biomineralization by activating autophagy, autophagic activator rapamycin and the autophagy inhibitor 3-MA were used to activate and inhibit autophagy, respectively. We then observed indicators of osteogenic differentiation and biomineralization of BMSCs. We found that both the activator group and the C_2_S NPs group showed increased expression of osteogenesis-related genes and proteins and promotion of mineralization and osteogenic differentiation. In the inhibitor group and inhibitor plus C_2_S NPs group, a downregulation of osteogenesis-related genes and proteins was observed, but compared with the inhibitor alone group, osteogenesis was induced in the inhibitor plus C_2_S NPs group, and the expression of genes and proteins related to osteogenesis was increased. These findings indicate that C_2_S NPs can improve the adverse effects of autophagy inhibition on osteogenic differentiation. The above experiments demonstrate that C_2_S NPs can promote osteogenic differentiation and biomineralization by promoting autophagy.

The signaling pathway that activates autophagy to promote osteogenesis was examined. AMPK is a signaling pathway that is involved in energy metabolism and has also been found to be involved in osteogenic differentiation. mTOR and ULK1, which are downstream of the AMPK pathway, are closely related to autophagy [38]. Studies have found that AMPK can directly or indirectly inhibit mTOR activation of the ULK1 pathway, while the Wnt/β-catenin pathway has been found to be related to osteogenic differentiation. We speculated that C_2_S NPs could activate autophagy through activation of the AMPK/ULK1 pathway and then activate the Wnt/β-catenin pathway to promote osteogenesis [39]. We verified the expression of related proteins in this pathway by Western blotting after coculture of C_2_S NPs and BMSCs. We found that C_2_S NPs could promote autophagy. Phosphorylation of the phagocyte-associated AMPK and ULK1 pathways and inhibition of mTOR phosphorylation promoted autophagy. This research additionally verified the expression level of β-catenin in the Wnt pathway and found that C_2_S NPs could promote the expression of β-catenin. We then detected the expression level of relevant proteins in this pathway using an autophagy agonist rapamycin and the autophagy inhibitors 3-MA. We found that the autophagy activator rapamycin and C_2_S NPs inhibited the phosphorylation of mTOR, upregulated the phosphorylation level of ULK1, and upregulated the expression of β-catenin using inhibitors, in contrast to the use of 3-MA. However, as observed for osteogenesis, the simultaneous use of inhibitors and C_2_S NPs could improve the effect of the inhibitor on the expression level of β-catenin. This phenomenon might be related to our observation that C_2_S NPs could be taken into the nucleus by cells, followed by direct activation of the transcription factor β-catenin and mediation of BMSC osteogenic differentiation [39]. Simultaneously, we also found that the expression of ULK1 was upregulated in vivo. The above results indicate that C_2_S NPs can further activate the Wnt/β-catenin pathway by activating the mTOR/ULK1 pathway and autophagy to promote osteogenesis and mineralization.

Our experiments showed that C_2_S NPs have good biocompatibility and biological activity and can be taken up by cells and promote proliferation, BMSC osteogenic mineralization, and differentiation. In vivo experiments also demonstrated that C_2_S NPs can promote new bone formation in the bone defect area. C_2_S NPs can provide raw materials for osteogenic mineralization of BMSCs without activating ALP to promote osteogenesis. The C_2_S NPs can enter the cell and be trafficked to autophagosomes to activate autophagy and further promote osteogenic differentiation of the MSCs. In vitro and in vivo experiments demonstrated that C_2_S NPs can activate the mTOR/ULK1 signaling pathway to induce autophagy and activate the Wnt/β-catenin pathway to promote osteogenesis. Therefore, C_2_S NPs can promote osteogenesis by activating autophagocytosis via the mTOR/ULK1 pathway.

## Conclusion

C_2_S NPs are a kind of bioceramic material with good biocompatibility that can promote osteogenic differentiation and biomineralization by activating autophagy. The mechanism underlying this biological effect consists of C_2_S NPs activation of the mTOR/ULK1 signaling pathway, induction of autophagy, activation of the Wnt/β-catenin pathway, and promotion of osteogenic differentiation and biomineralization.

## Supplementary information

**Additional file 1: Figure S1.** Biocompatibility evaluation of BMSCs exposed to C_2_S NPs (0, 10, 50, 100 μg/mL). a Uptake of C_2_S NPs by BMSCs was observed via TEM at 1, 3, and 7 days. C_2_S NPs shows in the boxes, autophagosomes shows in the arrow. b The levels of LDH release detected from BMSCs post-treatment with C_2_S NPs at 7 days. c Cells were stained with Annexin V-FITC and PI and analyzed by flow cytometry. Quantification of the cell early and total apoptosis ratio is shown below on the right. Values are expressed as the mean ± SEM. *n* = 3. *p < 0.05, **p < 0.01, ***p < 0.001 compared with the control group.

**Additional file 2: Figure S2.** a Surgical procedures in vitro. b Body weight change after treatment with C_2_S NPs for 4, 8, and 12 weeks. c Bone tissue stained with hematoxylin–eosin are treatment with C_2_S NPs for 4, 8, and 12 weeks. d Van Gieson’s picrofuchsin and Stevenel’s blue staining of newly formed bone in C2S NPs group at 4, 8, 12 weeks after operation.
