# Supplementary figures and images for: The mTOR/ULK1 signaling pathway mediates the autophagy-promoting and osteogenic effects of dicalcium silicate nanoparticles
Source: J Nanobiotechnology. 2020 Aug 31;18:119. doi: 10.1186/s12951-020-00663-w (PMC7457372; doi:10.1186/s12951-020-00663-w)

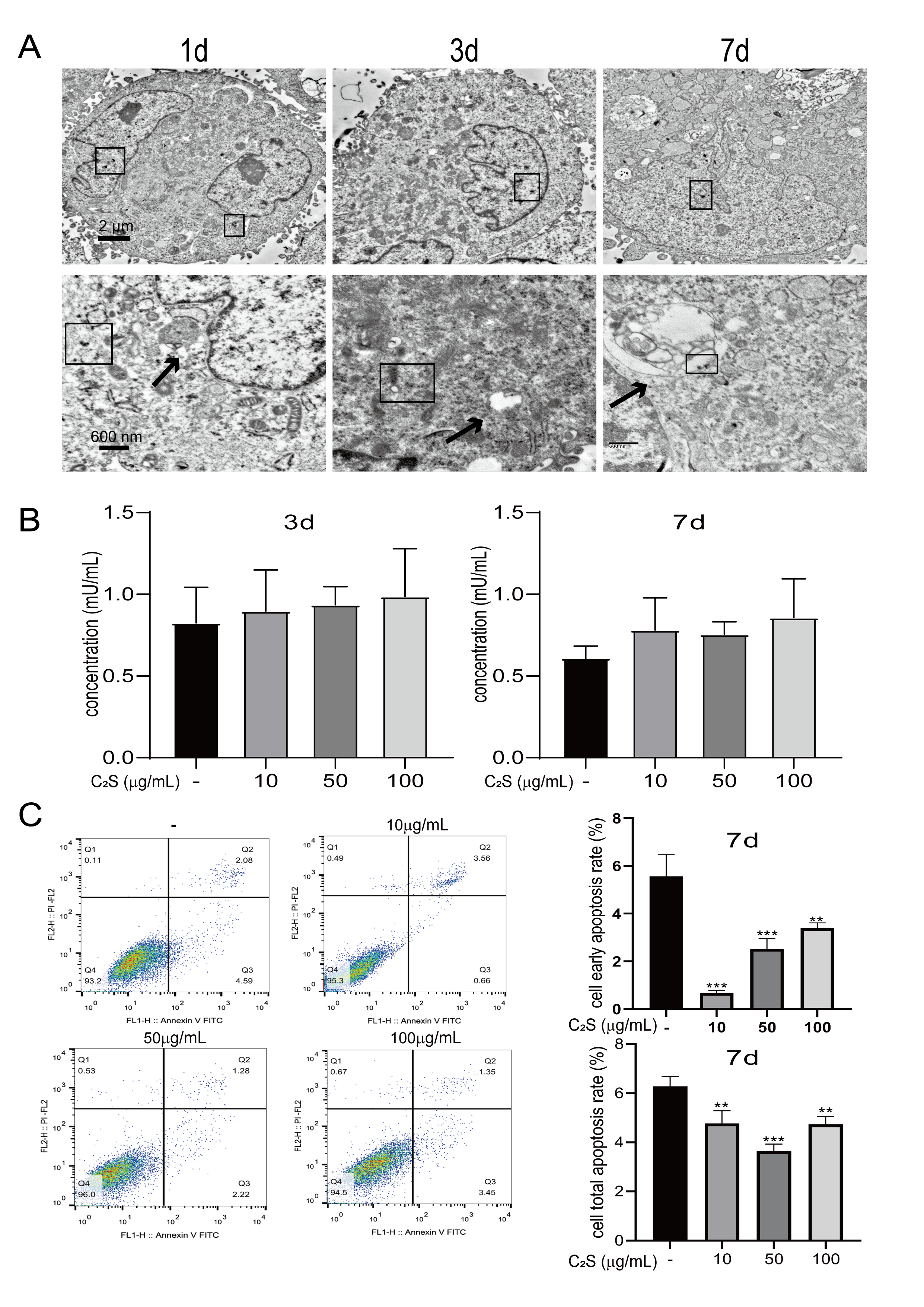

Supplement: Supplementary file 1 — Additional file 1: Figure S1. Biocompatibility evaluation of BMSCs exposed to C2S NPs (0, 10, 50, 100 μg/mL). a Uptake of C2S NPs by BMSCs was observed via TEM at 1, 3, and 7 days. C2S NPs shows in the boxes, autophagosomes shows in the arrow. b The levels of LDH release detected from BMSCs post-treatment with C2S NPs at 7 days. c Cells were stained with Annexin V-FITC and PI and analyzed by flow cytometry. Quantification of the cell early and total apoptosis ratio is shown below on the right. Values are expressed as the mean ± SEM. n = 3. *p < 0.05, **p < 0.01, ***p < 0.001 compared with the control group. [file 12951_2020_663_MOESM1_ESM.tif]

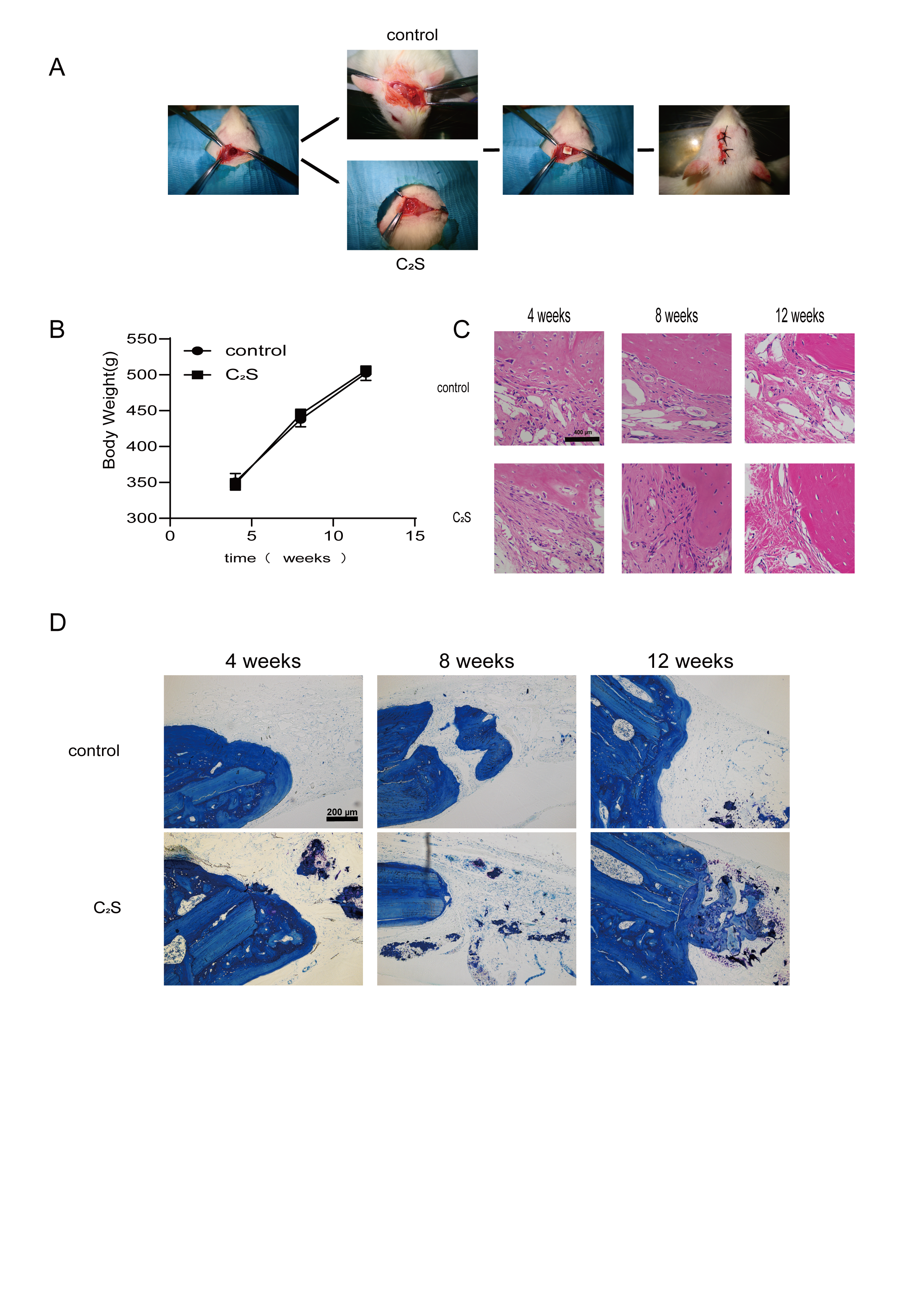

Supplement: Supplementary file 2 — Additional file 2: Figure S2. a Surgical procedures in vitro. b Body weight change after treatment with C2S NPs for 4, 8, and 12 weeks. c Bone tissue stained with hematoxylin–eosin are treatment with C2S NPs for 4, 8, and 12 weeks. d Van Gieson’s picrofuchsin and Stevenel’s blue staining of newly formed bone in C2S NPs group at 4, 8, 12 weeks after operation. [file 12951_2020_663_MOESM2_ESM.tif]
